# Optimized Dynamic Mode Decomposition via Non-Convex Regularization and Multiscale Permutation Entropy

**DOI:** 10.3390/e20030152

**Published:** 2018-02-27

**Authors:** Zhang Dang, Yong Lv, Yourong Li, Cancan Yi

**Affiliations:** 1 Key Laboratory of Metallurgical Equipment and Control Technology, Ministry of Education, Wuhan University of Science and Technology, Wuhan 430081, China; 2 Hubei Key Laboratory of Mechanical Transmission and Manufacturing Engineering, Wuhan University of Science and Technology, Wuhan 430081, China; 3 National Demonstration Center for Experimental Mechanical Education, Wuhan University of Science and Technology, Wuhan 430081, China

**Keywords:** dynamic mode decomposition, sparse optimization, non-convex regularization, multiscale permutation entropy, feature extraction

## Abstract

Dynamic mode decomposition (DMD) is essentially a hybrid algorithm based on mode decomposition and singular value decomposition, and it inevitably inherits the drawbacks of these two algorithms, including the selection strategy of truncated rank order and wanted mode components. A novel denoising and feature extraction algorithm for multi-component coupled noisy mechanical signals is proposed based on the standard DMD algorithm, which provides a new method solving the two intractable problems above. Firstly, a sparse optimization method of non-convex penalty function is adopted to determine the optimal dimensionality reduction space in the process of DMD, obtaining a series of optimal DMD modes. Then, multiscale permutation entropy calculation is performed to calculate the complexity of each DMD mode. Modes corresponding to the noise components are discarded by threshold technology, and we reconstruct the modes whose entropies are smaller than a threshold to recover the signal. By applying the algorithm to rolling bearing simulation signals and comparing with the result of wavelet transform, the effectiveness of the proposed method can be verified. Finally, the proposed method is applied to the experimental rolling bearing signals. Results demonstrated that the proposed approach has a good application prospect in noise reduction and fault feature extraction.

## 1. Introduction

Generally, amplitude-modulated and/or frequency-modulated (AM-FM) multi-component signals collected by sensors are interfered with by background noise, resulting lower signal-to-noise ratio (SNR) [1]. The reason for the low SNR signals obtained by a determinate acquisition system are usually as follows: firstly, when a rolling bearing is in the infantile fault period, the fault feature is inconspicuous and buried in the background noise. Secondly, signal energy as well as incipient failure features will attenuate because of the long distance between the fault source and the sensor, which usually is located on the bearing pedestal. Moreover, the characteristic signal will also be attenuated when there is a long transmission path from the sensor to the acquisition device [2]. Thirdly, the signals collected by sensors contain strong background noise. The main transmission system of a machine is a complex system composed of multiple components such as motors, couplings, multiple reduction gearbox. The vibration of all components will be collected by the sensor. Noisy signals make it difficult to identify the fault features in signal processing, therefore, it is necessary to perform denoising and characterize fault feature accurately on the low SNR vibration signal.

In the last few decades, many theoretical denoising methods have been proposed and are widely used in medical signaling, image processing and mechanical engineering signal processing [3,4,5]. Donolo and John-stone [6], taking the advantage of the spatial diversity of wavelet basis functions, proposed a denoising method with a threshold based on wavelet transform. Their methods concentrate the energy of the ideal signal, benefiting from the effective wavelet, guaranteeing a certain de-noising effect. However, the selection of threshold functions is the main problem with wavelet de-noising algorithms, which usually generate a deviation between the calculated result and the real one. Empirical mode decomposition (EMD) [7] can be treated as a typical adaptive signal decomposition method of frequency-selection and filtering. The signal is decomposed into a series of intrinsic mode functions (IMFs) arranged from high frequency to low frequency. The source signal can be processed by abandoning high-frequency components for the consideration that noise components are generally distributed in the high-frequency domain. However, as EMD has the defects of modal aliasing, the ideal signal and noise coexist in the selected IMFs. This would result in the loss of useful information. Ensemble empirical mode decomposition (EEMD) [8] was developed to solve the mode-mixing problem of EMD by adding noise to the source signal. It is more accurate and effective for data decomposition than EMD, but it prompts new trouble by being time consuming. Variable mode decomposition (VMD) [9,10] is a signal decomposition method based on wiener filtering, one dimensional Hilbert transform and heterodyne demodulation analysis, but the decomposition results are restricted by the selection of penalty parameters and the number of components. Singular value decomposition (SVD) [11], as a data-processing method without prior knowledge, has been successfully applied to signal denoising and proved to be effective. However, there exist two pivotal problems: one is that how to determine the effective rank order after decomposition, and the other one is that how to achieve the reconstruction matrix. Meanwhile, the noise reduction effect of SVD is not prominent when analyzing the low SNR signal.

Dynamic mode decomposition (DMD) [12] is an equation-free Koopman frequency analysis technique based on the theory of SVD and mode decomposition. It can extract the spatiotemporal coherent characteristics by decomposing a dynamic model into a series of single-frequency non-orthogonal modes based on its inherent space-time. The reconstructed signal can characterize the useful information of the original signal perfectly by superposing the filtered modes according to time coefficients. The algorithm is suitable for extracting dynamic information of nonlinear systems [13]. The instantaneous components and high-frequency noise components can be effectively removed, due to the dynamic delay processing in the algorithm [12]. The DMD algorithm has been well applied in the fluid mechanics community. Rowley [12] applied DMD in the jet cross flow field. The results show that DMD theory can extract the shedding frequency while the linear stability analysis method cannot. In addition, DMD can separate the shear layer and wall vortices, whereas the proper orthogonal decomposition (POD) algorithm is ineffective. Schmidt [13] applied DMD to the flow field in a variety of environmental conditions, indicating that the DMD theory is effective for feature extraction with numerical simulation and measured velocity field data. Moreover, DMD can separate video frames into a background and multiple foregrounds successfully when applied to video processing [14]. DMD can also be used for biometrics to detect fraudulent samples [15]. In the field of robotics and neuroscience, DMD has been used to estimate the perturbation of human-robot interactions [16], and the coherent modes in large-scale neural recording have been extracted [17]. Nowadays, theoretical research with DMD mainly focuses on two aspects. In the first aspect, a variety of improved methods are proposed based on the standard DMD to solve the problem that DMD cannot accurately extract the dynamic information due to the influence of signal noise. These strategies range from noise-corrected dynamic mode decomposition with control [18], total least-squares DMD [19], compressed sensing DMD [20] to sparsity-promoting DMD [21]. The improved DMD algorithm can extract fluid dynamic information more accurately in varying degrees. In the second aspect, in view of the large data processing problems, researchers aim at reducing the computational complexity and the program storage space in either pre-processing or post-processing, using strategies including random DMD [22], random low-rank DMD [23], and tensor-based DMD [24]. The standard DMD and its improved algorithm are more suitable in analyzing periodic or quasi-periodic signals, but it is prone to generate some false mode components. Therefore, the theory of fault vibration signal processing and fault mode recognition with DMD needs further study.

Essentially, DMD is a hybrid algorithm based on mode decomposition and SVD, and it inevitably inherits the drawbacks of these two algorithms, including the selection strategy of truncated rank order and DMD modes. The number of truncation rank for the similar matrix needs to be artificially and blindly selected in advance; whether the number value is appropriate or not is of crucial importance for accurately representing the dynamic information of reconstructed signal. The measured vibration signal with the characteristic of non-linear and non-stationary is coupled with multi-components and contain strong environmental noise. To achieve the purpose of fault feature extraction with DMD, it requires separation of the fault feature components effectively from the DMD modes. It is necessary to establish an adaptive optimization algorithm based on DMD to achieve an accurate truncation of rank and screen the single-frequency non-orthogonal modes effectively.

In this paper, an optimized DMD algorithm is proposed for extracting the fault features drowned in the multi-component coupled and noisy mechanical signal. The idea of sparse optimization is adopted to select the optimal rank for the solution of truncated rank order selection problem. By introducing the constraint of non-convex regulation to the rank function, the similar matrix has low rank attribute, making the ideal components (signals) more centralized. As for the fact that the noise’s multiscale permutation entropy (MPE) is larger than the ideal component’s, the low rank modes are selected corresponding to faults and inherent features by a threshold, solving the difficulty in selecting useful DMD modes. Then the signal is reconstructed by the low rank DMD modes, and Fourier transform (FT) is conducted to achieve the purpose of denoising and fault diagnosis of the noisy mechanical signal.

The structure of this paper is as follows. Section 2 introduces the basic theory, including the standard algorithm of DMD, the sparse optimization algorithm with non-convex optimization regulation, and MPE. In Section 3, the proposed algorithm is applied to the normal and faulty bearing noisy simulation signals to verify its effectiveness. Analysis results of two experimental bearing fault signals are described in Section 4. Conclusions are summarized in Section 5.

## 2. Methodology

In this part, the standard DMD algorithm is introduced based on time series in fault diagnosis. To solve the problem of selecting the optimal rank, a technique based on sparse optimization is proposed. The details are described as in Section 2.2. Then, we introduce the basic algorithm of MPE, and show the basic flow chart in the form of illustration.

### 2.1. DMD with Time Series Signal

Supposing that a signal S is acquired by numerical simulation or a sensor with equal intervals between two successive sampling points, $S=\left(x_{1},x_{2}\ldots ,x_{i},\ldots x_{N}\right),\;x_{i}\in R$, while $∆t=x_{i+1}-x_{i}$. Hankel matrix X can be obtained by a sliding window over the corresponding vector S.

(1) $$X=\left[\begin{array}{cccc} x_{1} & x_{2} & \cdots & x_{n}\\ x_{2} & x_{3} & \cdots & x_{n+1}\\ \cdots & & & \\ x_{i} & x_{i+1} & \cdots & x_{n+i-1}\\ \cdots & & & \\ x_{m} & x_{m+1} & \cdots & x_{N} \end{array}\right]=\left[X_{1},X_{2},\cdots ,X_{n}\right],X\in R^{m\times n}$$

where $X_{i}={\left(x_{i},x_{i+1}\ldots x_{s+i-1}\right)}^{T}$, m+n−1=N. Decomposing the Hankel matrix into two shift-stack matrices as in the form of Equation (2).

(2) $$X_{t}=\left[X_{1},X_{2},\cdots ,X_{n-1}\right];\;X_{t+1}=\left[X_{2},X_{3},\cdots ,X_{n}\right];\;X_{t},\;X_{t+1}\in R^{m\times (n-1)}$$

Assume that the two continuous sequence matrices satisfy a mapping relation with optimal linear operator. DMD algorithm utilizes a low rank expression of matrix A to capture the potential dynamic characteristics.

(3) $$X_{t+1}=AX_{t}$$

A can be also regarded as an operator to approximate the dynamic characteristics when the data are produced in nonlinear systems [13]. Matrix A usually contains many complex entries if *S* is presented in high-dimensional space. The DMD algorithm finds an optimal similarity matrix $F\in C^{r\times r}$ by projecting the matrix A with its *r*-th order eigenvectors according to POD.

(4) $$A=UFU^{*}$$

where $U^{*}$ is the complex conjugate transpose of POD mode U, and U is obtained by SVD of matrix $X_{t}$.

(5) $$X_{t}\approx U\Sigma V^{*}$$

where $U\in C^{m\times r},\;V\in R^{r\times (n-1)}$ are known as the left and right eigenvectors respectively, $U^{*}U=I,V^{*}V=I$, The symbol ∗ denotes the complex conjugate transpose. $\Sigma_{r}\in R^{r\times r}$ is a diagonal matrix containing *r* non-zero eigenvalues $\left\{\sigma_{1},\sigma_{2},\cdots \sigma_{r}\right\}$ on its diagonal.

The matrix F is determined by minimizing the Frobenius norm of the error between $X_{t+1}$ and $AX_{t}$.

(6) $$\Psi =\underset{F}{\arg\min}{\left\|X_{t+1}-UF\Sigma V^{*}\right\|}_{F}^{2}$$

Optimal solution of the above formula can be got directly.

(7) $$F_{DMD}=U^{*}X_{t+1}V\Sigma^{-1}$$

Equation (7) was proposed by Schmid [13], while the literature also introduced the application of DMD algorithm. Then a matrix $F_{DMD}\in R^{r\times r}$ with lower dimension is obtained. After the similar transformation above, $F_{DMD}$ and A are almost equivalent with each other. Hence, the eigenvectors and eigenvalues of A are replaced by the eigenvectors and eigenvalues of $F_{DMD}$.

Matrix $F_{DMD}$ optimizes the low-dimensional approximation of the internal data mapping matrix A by the POD subspace modes of $X_{t}$. The dynamic information of the *r*-dimensional subspace is expressed as:

(8) $$X_{t+1}=F_{DMD}X_{t}$$

Perform eigenvalue decomposition on the similarity matrix $F_{DMD}$:

(9) $$F_{DMD}=W\Lambda W^{-1}$$

where $W=[\omega_{1},\omega_{2},\cdots ,\omega_{r}]\in R^{r\times r}$ are the eigenvectors of similarity matrix $F_{DMD}$, and $\Lambda =diag([\lambda_{1},\lambda_{2},\cdots ,\lambda_{r}])\in R^{r\times r}$ is a diagonal matrix containing the corresponding complex eigenvalue $\lambda_{i}$.

The evolution of the signal characteristics can be characterized by similarity matrix. In addition, the *i*-th eigenvector of original operator is presented by relevant feature vector of similarity matrix.

(10) $$\phi_{i}=X_{t+1}V_{i}\Sigma_{i}^{-1}W_{i}$$

Equation (10), the project DMD approximate solution, is often called standard DMD mode. By firstly rewriting for convenience $\varpi_{i}=\ln(\lambda_{i})/∆t$, the approximate solution of reconstruction matrix of DMD is then given by:

(11) $$X_{DMD}=\sum_{i=1}^{r}\phi_{i}\exp(\varpi_{i}∆t)b_{i}=\Phi \exp(\Omega t)b,\;X_{DMD}\in R^{m\times n}$$

where Φ is a matrix consisted of DMD modes $\phi_{i}$, $\Omega =diag(\lambda_{i})$ is a diagonal matrix whose entries are continuous-time eigenvalues $\varpi_{i}$ of the similarity matrix $F_{DMD}$, b is a vector containing the initial amplitude of each mode, $b=\Phi^{\Gamma }x_{t}$, Γ denotes the Moore-Penrose pseudoinverse.

### 2.2. Sparse Optimized DMD via Non-Convex Regularization

As described in Section 2.1, *r*-th non-zero eigenvalues of the similarity matrix $F_{DMD}$ are retained to reconstruct the analytical signal, and *r* is the truncation rank order for the SVD of $X_{t}$. In the process of standard DMD, the rank of *r* needs to be manually preset. It plays a crucial role in accurately expressing the dynamic features of the original signal. If *r* is too large, it may reconstruct some unnecessary sparse components, such as noise, which cannot play a good denoising effect. Moreover, it increases the storage space and decreases the calculating efficiency. On the contrary, if *r* is too small, it will inevitably filter out useful low-rank components that symbolize the system dynamic information, failing to extract the signal physical characteristics. The difficulty lies in the determination of optimized truncation rank *r*.

Recently, many sparse optimization algorithms have been proposed in the fields of signal and image processing [16], compressive sensing [17], machine learning [25], and data mining [26] for consideration that the optimization variables have some sparse structures. Sparse optimization makes it possible to reconstruct high-dimensional signals and extract potential information from a small amount of data. Meanwhile, sparse optimization can greatly accelerate the calculation speed in large-scale optimization problems. Based on DMD theory, this paper introduces a parametric sparse optimization framework for the optimal low-rank approximation of $X_{t}$.

If the shift-stack Hankel matrix $X_{t}$ take the form of

(12) $$X_{t}=\tilde{X_{t}}+N_{t}$$

where $\tilde{X_{t}}$ is the ideal clean matrix containing all the dynamical characteristics of the vibration system. $N_{t}$ is a random noisy matrix. A typical sparse optimization framework with minimal low rank matrix approximation is defined as follows.

(13) $$X^{*}=\underset{F}{\arg\min}\{\frac{1}{2}{\left\|X_{t}-\tilde{X_{t}}\right\|}_{F}^{2}+\lambda \Pi \}$$

where λ>0 is a regularization coefficient, Π is a sparse regularization.

The above minimum low-rank approximation problem is transformed into a standard problem of minimizing nuclear penalty when $\Pi =\sum_{i=1}^{k}\left|\sigma_{i}\left(X_{t}\right)\right|$. Theoretically, the nuclear norm optimization framework is convex, and it can be directly solved by taking traditional SVD on the shift-stack Hankel matrix $X_{t}$. However, the solution of convex norm-based nuclear norm optimization often leads to underestimate the rank of the original matrix [27]. Selesnick [28] proposed a sparse optimization method based on non-convex regularization to denoise a noisy signal. The calculation process can guarantee the strict convexity of Equation (13) and generates a unique solution. Inspired by the idea of [28,29], this paper adopts a sparse framework of non-convex optimization to obtain the best truncated rank of the shift-stack matrix. Non-convex optimization can approximate the non-zero singular value of the calculation matrix more accurately than nuclear norm optimization framework. Moreover, the non-convex method is more robust to strong background noise then nuclear norm optimization.

Let us define $\Pi =\sum_{i=1}^{k}\phi (\sigma_{i}(X_{t});\alpha )$, ϕ:R→R corresponding to the non-convex sparse regularization of sparse optimization. Non-convex penalty function must meet the following five properties:

(1) ϕ is symmetric and continuous in *R* space. i.e., ϕ(−x;a)=ϕ(x;a), which is continuous derivable in R;
(2) $\phi^{\prime }(x;a)>0,\;\forall x>0;$
(3) $\phi^{\prime \prime }(x;a)\leq 0,\;\forall x>0;$
(4) $\phi^{\prime }(0^{+};a)=1;$
(5) $\underset{x\neq 0}{\inf}\phi^{\prime \prime }(x;a)=\phi^{\prime \prime }(0^{+};a)=-a;$

Chen [30] listed various penalty functions satisfying the above assumption in Table II. Here we employ the partly quadratic penalty function [27], defined as:

(14) $$\phi (x;a)=\left\{ \begin{array}{ll} \left|x\right|-\frac{a}{2}x^{2}, & \left|x\right|<\frac{1}{a}\\ \frac{1}{2a}, & \left|x\right|\geq \frac{1}{a} \end{array}\right.$$

where α is a non-convex penalty parameter and controls the degree of non-convexity of ϕ, satisfying $0\leq \alpha \leq \frac{1}{\lambda }$.

Finally, the sparse optimization objective function based on non-convex regularization is defined as Equation (15). The optimal truncation rank *r* of singular value which should be determined in the process of DMD algorithm is obtained by the non-convex optimization framework.

(15) $$X^{*}=\underset{F}{\arg\min}\{\frac{1}{2}{\left\|X_{t}-\tilde{X_{t}}\right\|}_{F}^{2}+\lambda \sum_{i=1}^{k}\phi (\sigma_{i}(X_{t});\alpha )\}$$

### 2.3. Optimized DMD Modes via MPE

The result of the DMD algorithm obtains a series of single-frequency modes that represent the dynamics of the original system. DMD modes are similar to the IMFs obtained by EMD, while each DMD mode corresponds to a single frequency. It can also be regarded as the vibration modes in mechanical vibration. All DMD modes express the inherent characteristics, fault information and noise background components (introduced by the shift-stack matrix $X_{t+1}$) of the original system information, making it difficult to diagnose mechanical fault features effectively. Approximate entropy (AE) [31] reflects the time series’ complexity by calculating the approximate degree of two points in the given dimension and the probability of new mode when the dimension changes. AE has the characteristics of stable calculation results with short data sequence. Sample entropy [32,33] is an improved algorithm, and it performs better than AE to maintain the consistency of the calculation results. Permutation entropy (PE) [33] is an algorithm based on time series phase space reconstruction and neighborhood value comparison, which can effectively describe the complexity of the system. PE has the advantages of simple algorithm, fast computing speed, and reliable calculation results [34]. MPE is an improved algorithm based on PE. In addition, it is especially suitable for nonlinear data. In this paper, MPE threshold is used to identify and remove the modes of noise components. The remaining low-rank components are reconstructed by DMD to achieve the purpose of noise reduction and fault feature extraction for noisy fault signals.

The basic idea is that it first gets the time series coarse-grained in time-scale, then calculates PE on the coarse-grained data. The process of MPE is briefly described here. For more detailed information about MPE, it can be referred to in [33,35].

First, a given time series $\left\{x_{k},k=1,2,\cdots ,N\right\}$ is coarse-grained.

(16) xjs=1s∑i=(j−1)s+1jsxk (1≤j≤[Ns])

where S is the scale factor, S=1,2,…, when S=1, the coarse-grained sequence is the original sequence, and the result of MPE is PE. [Ns] means rounding Ns. If S≥2, the original sequence is coarse-grained with the length of [Ns].

Secondly, the phase space reconstruction is carried on the coarse-grained time series.

(17) $$x_{i}^{d}=[x_{\left(i\right)},x_{\left(i+\tau \right)},\cdots ,x_{\left(i+(m-1)\tau \right)}]$$

where i=1,2,⋯,N. d≥2 is the embedding dimension; τ is the delay time; Each row vector in the reconstructed phase space matrix is a reconstruction vector, and there are d reconstruction vectors in total.

Considering every two data in the form of ascending or descending order, the j-th reconstruction vector has d! possible ordinal patterns. Finally, the MPE value H is defined as:

(18) $$H(N,d,\tau )=-\frac{1}{\ln(d!)}\sum_{i=1}^{d!}\frac{p_{i}}{N}\ln(\frac{p_{i}}{N})$$

where $p_{i}$ represents the frequency of a particular sequence pattern in all reconstruction vectors.

**Figure 1:**
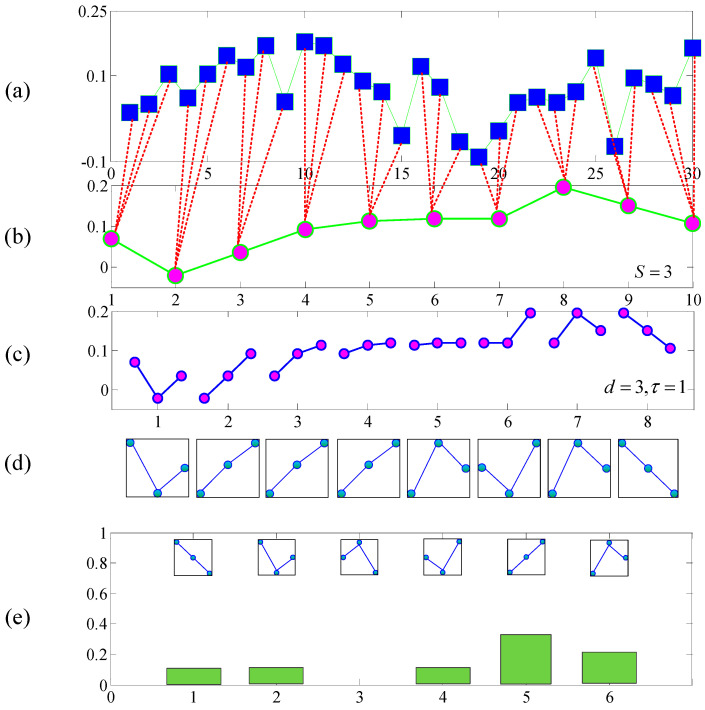
An example for the schedule of calculating $p_{i}$ with MPE. (**a**) noisy time series with length of N=30; (**b**) coarse-grained time series within non-overlapping time slice referred to as the scale factor s=3; (**c**) data segments with embedded dimension d=3 and time delay τ=1; (**d**) ordinal patterns according to the data segments; (**e**) The frequency of each particular ordinal patterns of all the data segments.

Figure 1 shows the calculation process of $p_{i}$ with a few capacity sample data sequences. The regular parameter ln(d!) ensures that the maximum value of H(N,d,τ) is 1.

MPE is a measure of system complexity. The larger the entropy, the more complex the system components, and the closer the system is to the stochastic signal. Conversely, the smaller the entropy value, the simpler the system components appears. For the fixed-length analysis sequence, the result of MPE dependent on the scale factor s and the embedding dimension d. Generally, if d is too small, the reconstructed vector contains little information, the algorithm loses its validity and cannot detect the kinetic mutation of the sequence. If d is too large, the reconstruction of phase will homogenize the time series, which is not only time-consuming but also unable to reflect the small changes of the sequence. Bandt [33] and Zunino [34] suggested that d should be in the range 3–7. Time delay τ has little effect on the MPE of time series [35,36], and in most literatures [36,37,38], authors employ τ=1. In this paper, we employ s=5, d=6, τ=1 in the calculation of MPE. In [36,38,39], the length *N* of time series are suggested as N>5d!, thus the sample points of the simulation signal should be larger than 3600.

**Figure 2:**
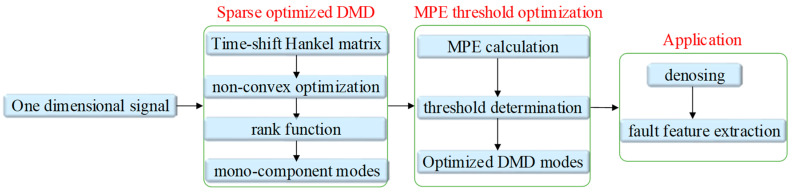
A flow chart of the proposed algorithm.

Ultimately, the flowchart of the algorithm based on DMD via non-convex regularization and MPE is shown in Figure 2.

## 3. Algorithm Application in Fault Bearing Simulation Signal

Here we adopt the dynamic model of bearing fault signal established by Randall [40]. The mathematical model of fault rolling bearing is described in Equation (19). According to the characteristics of bearing structure, instantaneous impact signal should be generated when bearing parts pass through the fault location. The impact motivates the bearing system oscillating by its natural frequency with high-frequency attenuation. Define T as a period of two intervals, S(t) as the natural frequency oscillation function, and $A_{k}$ as the i-th amplitude of shock response. The interference of additive noise $n_{t}$ (zero mean additive background noise) should be taken into consideration in the simulation signal in view of poor working environment of rolling bearings. Therefore, the mathematical model of fault rolling bearing can be described as:

(19) $$\{\begin{array}{l} f(t)=\sum_{k=1}^{M}A_{k}S(t-kT-\tau_{k})+n(t)\\ A_{k}=a_{k}\cos(2\pi f_{m}t+\varphi_{A})+c_{A}\\ S(t-kT-\tau_{k})=\exp[-B(t-kT-\tau_{k})\cdot \sin[2\pi f_{n}(t-kT-\tau_{k})+\gamma ] \end{array}$$

where $a_{k}$ is the *k*-th impact energy; γ and $\varphi_{A}$ are the initial phases; $f_{m}$ is the modulation frequency; B is the attenuation coefficient depended on the bearing system; $\tau_{k}$ is the time lag from its mean period due to the presence of tiny fluctuation, and $c_{A}$ is a random constant.

**Figure 3:**
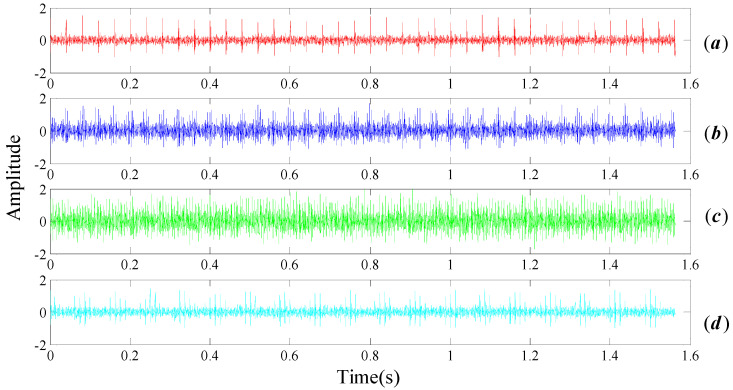
Time domain simulation signals of rolling bear including noise with SNR 0.5 (**a**) normal bearing; (**b**) inner fault; (**c**) outer fault; (**d**) cage fault.

Time domain simulation signals of rolling bearing with normal bearing and inner race, outer race and roller faults are respectively plotted in Figure 3. It should be pointed out that Gaussian white noise is added so as to SNR reach to 0.5 precisely. The parameters selection of Equation (19) is listed in Table 1, where $f_{r}$ and $f_{c}$ are denoted as rotational frequency and cage frequency. $f_{i}$, $f_{o}$, $f_{b}$ respectively represent the failure frequency of the inner circle, the outer ring, and the rolling element. The sampling frequency is selected as 5 KHz, and the number of total sampling points is 8000.

**Figure 4:**
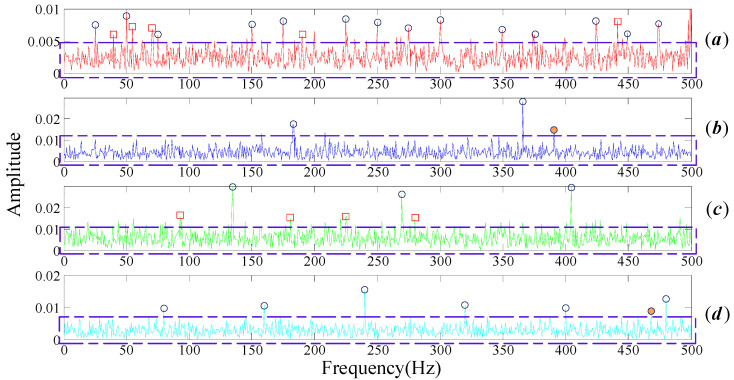
Frequency spectrum of rolling bear simulation signals with noisy (**a**) normal bearing; (**b**) inner fault; (**c**) outer fault; (**d**) cage fault.

The FT spectrum analysis of the bearing noisy signal is shown in Figure 4. There are a large number of noisy spectral components, as shown by the blue dotted boxes in Figure 4a–d, which severely affect the feature identification of the fault. Rotational frequency and its resonance frequencies (as marked in blue circles in Figure 3a) should be easy to recognize in the spectrum domain when the bearing is under healthy conditions. However, numbers of noise frequencies revolve around the center frequencies, as marked in the brown dotted box in Figure 3a, resulting in the fact that we cannot interpret the signal properly. The same situation appears in the bearing inner race fault signal spectrum, as shown in Figure 3c. In general, the three common types of faults on the inner race, the outer race and the rolling elements can be modeled as $f_{m}=f_{r}$, $f_{m}=0$, $f_{m}=f_{c}$, respectively. That is to say, rotational frequency, 0, cage frequency should modulate their center frequency, respectively. In Figure 3b,d, the center frequency belongs to the inner race and balls can be identified, but it is difficult to identify their respective modulation frequency as they are submerged by the noise components.

**Figure 5:**
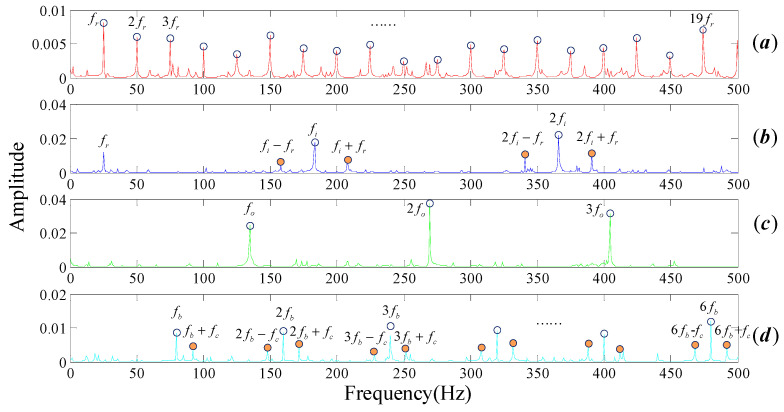
Frequency spectrum of the noisy signals provided by the proposed algorithm. (**a**) Normal bearing; (**b**) inner fault; (**c**) outer fault; (**d**) cage fault.

In this paper, the parameters λ, a are selected as 1.2, 0.5 respectively in the process of sparse optimization using non-convex optimization regularization. The MPE threshold is selected as 0.7 (refer to the conclusion section). We recover the signals by DMD modes whose MPE values are less than the threshold. Spectrums were obtained by performing FT on the reconstruction signals, as shown in Figure 5.

**Figure 6:**
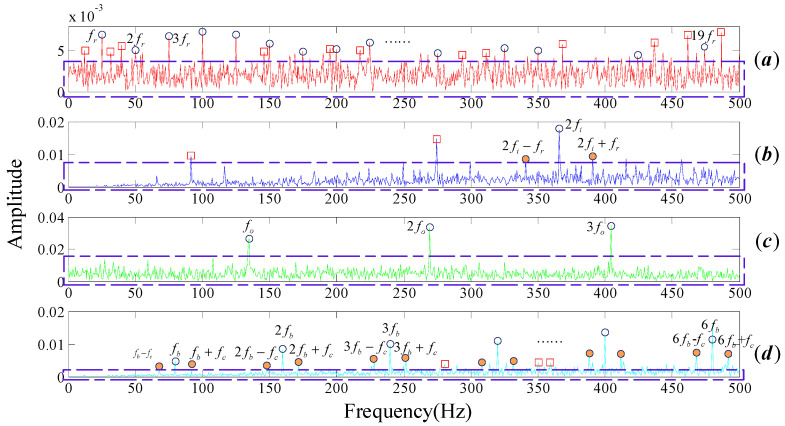
Frequency spectrum of the noisy signals provided by WT. (**a**) Normal bearing; (**b**) inner fault; (**c**) outer fault; (**d**) cage fault.

Meanwhile, in order to illustrate the effectiveness of our method, multi-scale wavelet transform is applied on the four noisy signals by taking the wavelet function db1, WT results are shown in Figure 6. Though it is obvious that the harmonic frequencies of $f_{r}$ and $f_{o}$ can be identified in Figure 6a,c, there are still some frequencies attributed by the noise involving around the mid-frequencies, as can we see in the brown dotted box. Meanwhile the spectra of the proposed algorithm are cleaner and more readable then the results of FT and WT. Not only can we pick out their center and modulation frequencies in Figure 5, but also there is few noise frequencies surrounding them. Our method has tremendous advantages in both de-noising and fault feature extraction compared with FT and WT.

## 4. Algorithm Application in Experimental Signals of Rolling Bear

Signals of bearing systems collected by sensors from practice spots are much more complex than the simulation signals, which contain multi-component signals and background noises transmitted by other components. In order to verify the effectiveness of the proposed algorithm in signal de-noising and fault diagnosis, we apply the proposed algorithm to the actual bearing signals. We test the bearing signals from two experimental environments. First, the bearing signal is from the published bearing test data at the university of Cincinnati [41]; the second comes from our rotating machinery vibration analysis test rig.

**Figure 7:**
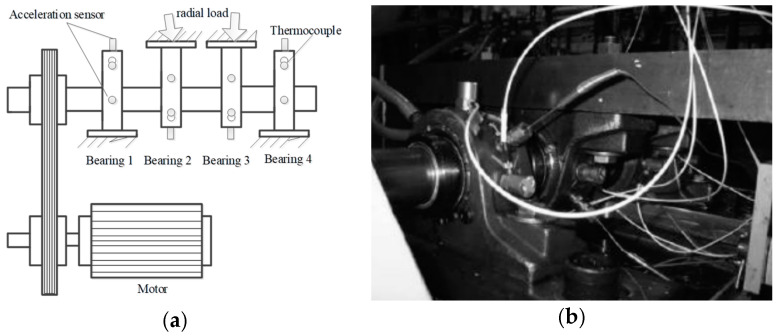
The Intelligent Maintenance Systems of Cincinnati: (**a**) the schematic diagram of the bearing test rig; (**b**) spot photo of the bearing test rig.

The Intelligent Maintenance Systems of Cincinnati is shown in Figure 7. Four Rexnord ZA-2115 double row bearings were installed on a shaft, which was driven by an AC motor at the speed of 2000 RPM via sever rub belts. Each row of the bearing has Z=16 roller elements. The circle diameter of the bearing bitch and roller elements are respectively D=28.15 mm, d=3.31 mm, and the bearing’s contact angle is α = 15.17°. They utilized high sensitivity quartz ICP accelerometers (PCB 353B33), installed on the bearing housing, collecting the vibration signals and NI DAQ Card 6062E collecting the signals.

We take their experimental data of fourth channel in Set No. 3 to verify our method. The total number of the time series data is 20,480, and the sampling frequency is 20 KHz. Finally, the bearing failed because of outer race fault. The rotation frequency and outer ring fault frequency can be respectively calculated as $f_{r}=33.33\;\mathrm{Hz},\;f_{o}=236.17\;\mathrm{Hz}$ by formulas [42].

**Figure 8:**
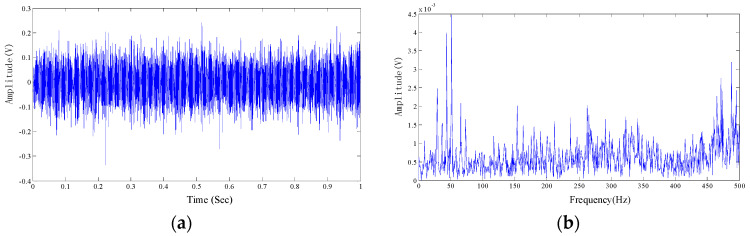
The outer race fault signal of the Intelligent Maintenance Systems of Cincinnati in time domain and frequency: (**a**) Time domain; and (**b**) Frequency domain of FT.

The time domain and the frequency domain of the selected signals are shown in Figure 8. Obviously, it is hard to identify whether there is a bearing fault from Figure 8b for such large amounts of interference frequency presented in the frequency spectrum.

**Figure 9:**
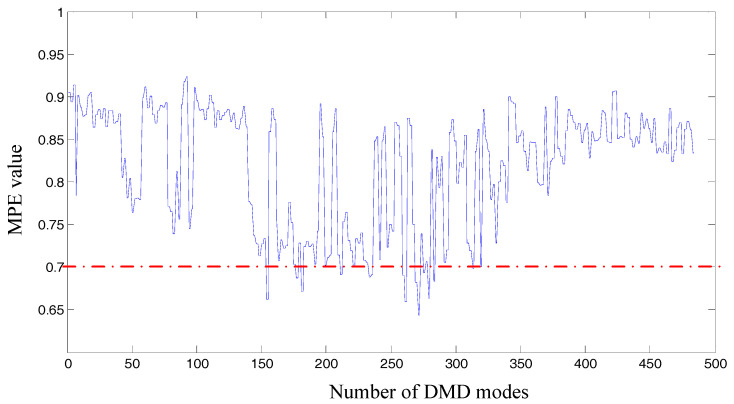
MPE values of DMD modes, the original signal is from Cincinnati.

**Figure 10:**
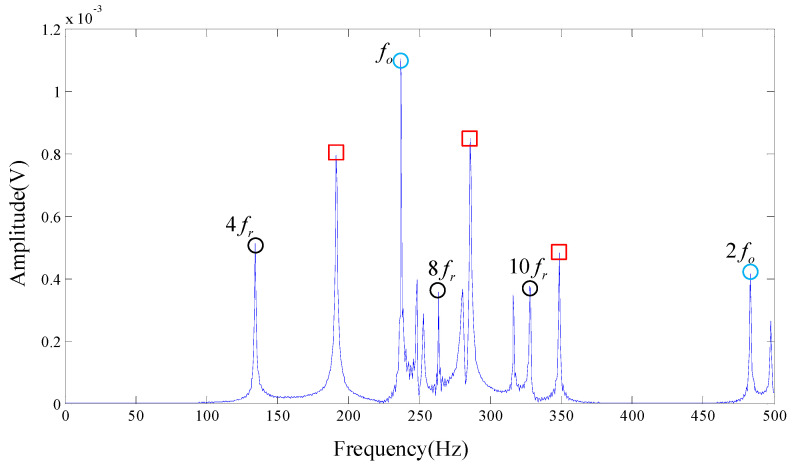
The result provided by our algorithm, the original signal is from Cincinnati.

Then we apply our algorithm on the chosen signal with chosen parameters λ=1.2, a=0.5, s=5, d=6, τ=1. The truncation rank is calculated as 484 by applying non-convex optimization regularization on the rank function. The same number of single-frequency DMD modes are obtained correspondingly. The MPE values corresponding to each DMD modes are shown in Figure 9. We recover the wanted signals by DMD modes whose MPE values are less than 0.7. Spectrums are obtained by performing FT on the reconstruction signal, as shown in Figure 10.

As shown in Figure 10 there are a few characteristic frequencies located in the frequency spectrogram provided by our algorithm. Outer race failure frequency $f_{o}$ and its resonant frequency $2f_{o}$ can be easily captured. Also, some resonant frequencies of the rotation frequency $f_{r}$ are presented in the spectrum. However, regrettably, some of the frequencies we cannot account for are still in Figure 10, which are shown in the red box, revealing that the signal of the actual bearing system is complex. Yet we still got the failure frequency of the bearing test signal, which is in line with experimental results.

**Figure 11:**
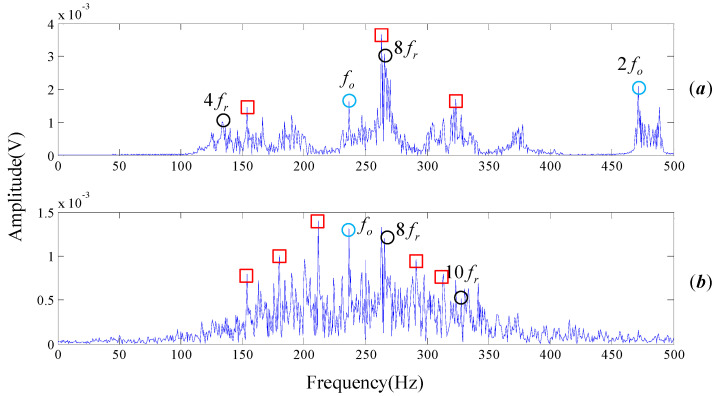
Frequency spectrogram provided by wavelet packet decomposition and EEMD. (**a**) Results of wavelet packet decomposition; (**b**) Frequency spectrogram of IMF3 decomposed by EEMD.

Wavelet packet decomposition and EEMD are applied on the experimental signal, as shown in Figure 11. Figure 11a is the spectrum of the recover signal transformed by wavelet packet, with db15 and the default threshold. The optimal amplitude of the noise is set as 0.2 and the ensemble size is 100 for the EEMD. Figure 11b shows the spectrum of IMF3 which has the largest correlation with the original signal. A large amount of unexplainable interference frequencies, visible in the spectrogram, seriously affect the recognition of the rotation frequency and outer ring fault frequency. Thus, the optimized DMD outperforms wavelet packet decomposition and EEMD in the mode decomposition of rolling bearing experimental signal.

**Figure 12:**
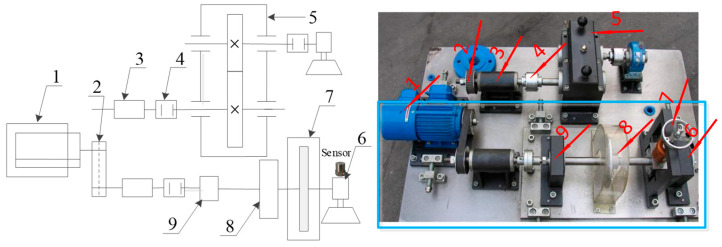
Rotating machinery vibration analysis test rig. (**a**) Structure diagram of test device; (**b**) physical object of the device; 1—motor, 2—belt pulley, 3—drive shaft support, 4—coupler, 5—gear box, 6—bearing pedestal, with replaceable rolling bearing NU205, 7—belt pulley, 8—radial force device, 9—bearing pedestal, with rolling bearing not replaced.

**Figure 13:**
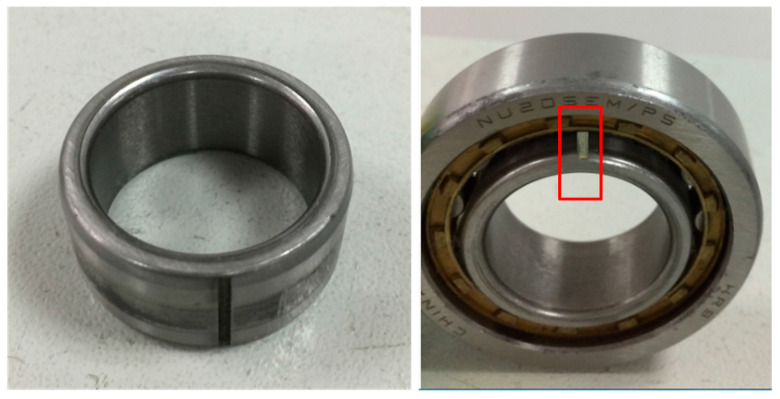
Physical diagram of the fault bearing.

Next, we apply the algorithm on the experimental signal measured by our rotating machinery vibration analysis test rig. The physical structure of the experimental rig is shown in Figure 12. The whole device consists of a variable speed drive motor, shaft, gear box, deflection wheel, bearing and governor. A picture of the sensor in Figure 12 refers to the position where the single channel vibration acceleration sensor (PCB-352C33, NY, USA) is installed. A cylinder roller bearing NU205 with artificial defects on the outside surface of the inner race was mounted in the housing. The fault bearing physical diagram is shown in Figure 13. The number of rolling elements is Z=13, and the cylinder roller diameter is d=7.5 mm, pitch diameter of the testing bearing is D=39 mm. We set the motor at the speed of 980 RPM. So, rotation frequency and outer ring fault frequency can be calculated as $f_{r}=16.3\;\mathrm{Hz},\;f_{i}=126.3\;\mathrm{Hz}$, respectively [42].

**Figure 14:**
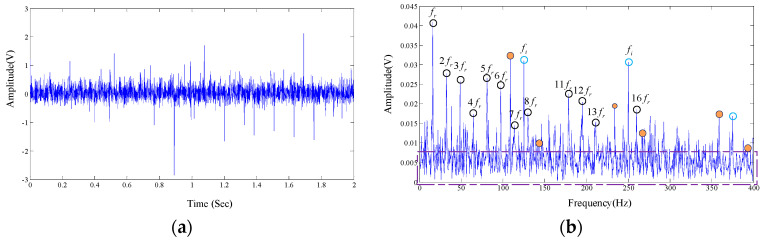
The measured signal in time domain and frequency, the original signal is from our test rig. (**a**) Time domain; and (**b**) Frequency domain of FT.

A total number of 4000 sample points, shown in Figure 14a in time domain, are taken into analysis with the proposed algorithm. The frequency domain is shown in Figure 14b.

As is shown in Figure 14b, rotational frequency and its resonant frequencies are presented in the spectrum diagram. The center frequency of the inner race can also be easily found out, but the modulation components are not obvious. Meanwhile, there are large amounts of interference frequency presented in the frequency spectral. The spectrum of the test signal is not intuitionistic, which makes it difficult to diagnose the fault in view of the technicians.

**Figure 15:**
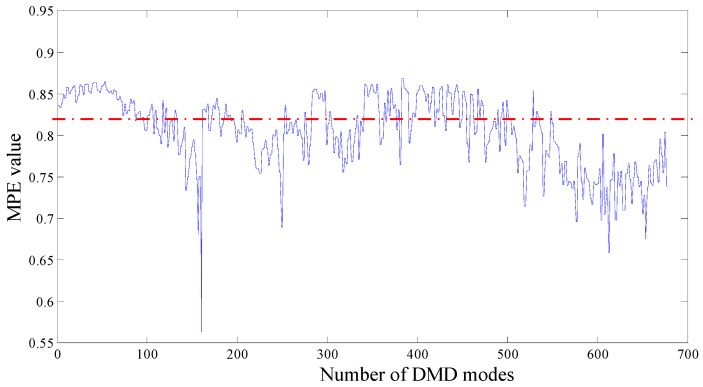
MPE values of DMD modes, the original signal is from our test rig.

**Figure 16:**
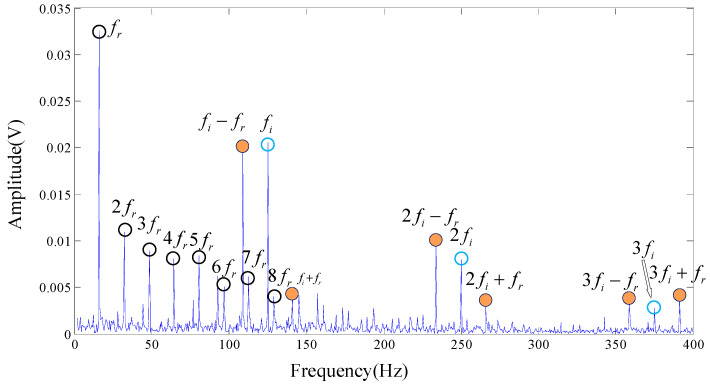
The result provided by the proposed algorithm, the original signal is from our test rig.

Subsequently, the proposed algorithm is applied to the tested signal with the same parameters λ=1.2, a=0.5, s=5, d=6, τ=1. 682 single-frequency DMD modes are obtained by sparse optimization algorithm. Figure 15 shows the MPE values of each DMD modes. DMD modes whose MPE values are less than 0.82 are reconstructed to form a recovery signal. Spectrums are obtained by performing FT on the reconstruction signal, as shown in Figure 16.

**Figure 17:**
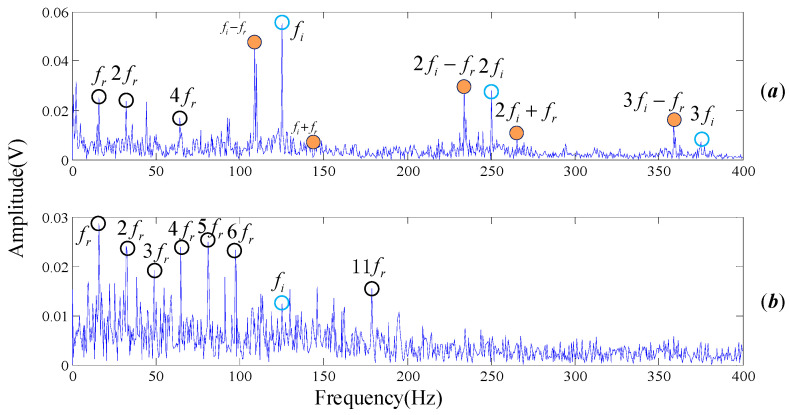
Frequency spectrogram provided by wavelet packet decomposition and EEMD. (**a**) Results of wavelet packet decomposition; (**b**) Frequency spectrogram of IMF1 decomposed by EEMD.

Wavelet packet decomposition and EEMD are also employed on our experimental signal, as shown in Figure 17. The methods and parameters used in the analysis are consistent with the signal processing procedure of Cincinnati. Here the spectrum of IMF1 decomposed by EEMD is selected because it has the largest correlation with the original signal, as shown in Figure 17b. Obviously, Figure 16 is more readable than Figure 13b and Figure 17. The rotational frequency $f_{r}$ and its harmonics ($2f_{r}$, $3f_{r}$, $4f_{r}$ $5f_{r}$, ⋯) are easily identified. Not only could the center frequency be pointed out clearly but also the modulation frequency of the inner face. That is to say, we are more confident to judge that the inner race of the testing rolling bearing has encountered failure, which is highly consistent with the actual situation. The basic reason the frequencies are easy to read is that there are a few interference frequencies nearby them. Therefore, the proposed algorithm of noise reduction and feature extraction is proved to be practical and to outperform other mode decompositions such as wavelet packet decomposition and EEMD.

## 5. Conclusions

As a powerful tool for Koopman spectrum analysis technology with the characteristic of equation-free and data driven, DMD decomposes the time series into a series of single-frequency modes without any assumption. Theoretically, dynamic features of the original system including fault features and noise components exist in these DMD modes only if the truncation rank order *r* is appropriate. The truncated rank order needs to be preset artificially in the standard DMD algorithm, which is a crucial problem. Another puzzle with the application of standard DMD is of how to define a selection strategy to filter the undesired DMD modes in the process of signal reconstruction.

By proposing solutions for the selection strategy of truncated rank order and DMD modes, this paper puts forward a novel denoising and feature extraction algorithm for multi-component coupled noisy mechanical signals. Non-convex penalty regularization is adopted to the rank function of the shift-stack matrix, and a series of optimal DMD modes are obtained by this sparse optimization method. MPE is performed as a tool of optimal mode selection. After calculating the MPE of each DMD mode, signal is reconstructed by the modes whose entropies are smaller than a threshold. The proposed algorithm is successfully applied in rolling bearing simulation and experimental signals, demonstrating that it has a good application prospect in noise reduction and fault feature extraction.

DMD algorithm has been widely used in the field of fluid mechanics and video processing. This paper presents an optimized DMD algorithm and applies it to mechanical vibration signal. The parameters of non-convex penalty regularization and MPE in our algorithm are proved to be appropriate by repeated verification. The non-convex optimization method is used to extract the dynamic characteristics of the original system more comprehensively than selecting truncation rank with a “elbow” of the singular value. The dynamic characteristics, fault features and noise components of the original signal can be identified by using MPE method. However, for filtering the noise components, entropy threshold needs to be set by multiple attempts in our algorithm. For example, when dealing with the experimental signal of Cincinnati, we firstly set the threshold values of 0.7, 0.75, 0.8, etc., and then observed the effect of the recovery signal through the spectrum diagram respectively. We found that when the threshold value was 0.7, the fault characteristics were more obvious, and the interference frequencies were less. Therefore, 0.7 was selected as the criterion. Research on different vibration signals, various transmission systems and different degrees of fault damage to select a certain MPE threshold is our future work.

## Figures and Tables

**Table 1:** The parameters selection with simulation signals of rolling bearing.

$a_{k}$	γ	$\varphi_{A}$	B	$\tau_{k}$	$c_{A}$	$f_{r}(\mathrm{Hz})$	$f_{i}(\mathrm{Hz})$	$f_{o}(\mathrm{Hz})$	$f_{b}(\mathrm{Hz})$	$f_{c}(\mathrm{Hz})$
3.5	0	0	750	0.01	1	25	180	130	80	12
